# The role of opioids in cancer response to immunotherapy

**DOI:** 10.1186/s12967-021-02784-8

**Published:** 2021-03-23

**Authors:** Andrea Botticelli, Alessio Cirillo, Giulia Pomati, Bruna Cerbelli, Simone Scagnoli, Michela Roberto, Alain Gelibter, Giulia Mammone, Maria Letizia Calandrella, Edoardo Cerbelli, Francesca Romana Di Pietro, Federica De Galitiis, Gaetano Lanzetta, Enrico Cortesi, Silvia Mezi, Paolo Marchetti

**Affiliations:** 1 Department of Clinical and Molecular Medicine, Sapienza University of Rome, 00185 Rome, Italy; 2 Department of Radiological, Oncological and Pathological Science, Sapienza University of Rome, 00185 Rome, Italy; 3 Department of Molecular Medicine, Sapienza University of Rome, Viale Regina Elena 291, 00161 Rome, Italy; 4 Department of Medical and Surgical Sciences and Translational Medicine, University of Rome Sapienza, 00185 Rome, Italy; 5 Istituto Dermopatico Dell’Immacolata, IDI-IRCCS, 00167 Rome, Italy; 6 Medical Oncology Unit, Italian Neuro-Traumatology Institute, 00046 Grottaferrata, Italy

**Keywords:** Immunotherapy, Opioids, Opioid receptors, Prognostic factor, Predictive factor, Early progression

## Abstract

**Background:**

The response to immunotherapy can be impaired by several factors including external intervention such as drug interactions with immune system. We aimed to examine the immunomodulatory action of opioids, since immune cells express opioid receptors able to negatively influence their activities.

**Methods:**

This observational, multicenter, retrospective study, recruited patients with different metastatic solid tumors, who have received immunotherapy between September 2014 and September 2019. Immunotherapy was administered according to the standard schedule approved for each primary tumor and line of treatment. The concomitant intake of antibiotics, antifungals, corticosteroids and opioids were evaluated in all included patients. The relationship between tumor response to immunotherapy and the oncological outcomes were evaluated. A multivariate Cox-proportional hazard model was used to identify independent prognostic factors for survival.

**Results:**

One hundred ninety-three patients were recruited. Overall, progression-free survival (PFS) and overall survival (OS) were significantly shorter in those patients taking opioids than in those who didn’t (median PFS, 3 months vs. 19 months, HR 1.70, 95% CI 1.37–2.09, *p* < 0.0001; median OS, 4 months vs. 35 months, HR 1.60, 95% CI 1.26–2.02, *p* < 0.0001). In addition, PFS and OS were significantly impaired in those patients taking corticosteroids, antibiotics or antifungals, in those patients with an ECOG PS ≥ 1 and in patients with a high tumor burden. Using the multivariate analyses, opioids and ECOG PS were independent prognostic factors for PFS, whereas only ECOG PS resulted to be an independent prognostic factor for OS, with trend toward significance for opioids as well as tumor burden.

**Discussion:**

Our study suggests that the concomitant administration of drugs as well as some clinical features could negatively predict the outcomes of cancer patients receiving immunotherapy. In particular, opioids use during immunotherapy is associated with early progression, potentially representing a predictive factor for PFS and negatively influencing OS as well.

**Conclusions:**

A possible negative drug interaction able to impair the immune response to anti-PD-1/PD-L1 agents has been highlighted. Our findings suggest the need to further explore the impact of opioids on immune system modulation and their role in restoring the response to immunotherapy treatment, thereby improving patients' outcomes.

## Background

The immune-checkpoint monoclonal antibodies inhibitors (ICIs), a class of drugs targeting the inhibitory immune-checkpoint receptors, have demonstrated significant improvement in overall survival (OS) in many cancer types and actually representing a revolutionary milestone in oncology [1]. The immune system is involved in the recognition and destruction of cancer cells, nevertheless tumor subclones with reduced immunogenicity, such as loss of antigen presentation, low levels of programmed death ligand-1 (PDL1) expression and IFN-γ secretion by T cells, can be selected^2^ avoiding immune destroy and leading to tumor growth and clinically evident disease [2, 3].

Several studies have demonstrated that, in a proportion of patients, ICIs can induce durable response, generating long-lasting specific immunological memory against tumor [4]. Thus, immunotherapy has become the standard of care in several solid tumors, including advanced melanoma [5, 6], no-small cell lung cancer (NSCLC) [7–10], renal cell carcinoma (RCC) [11, 12], Merkel carcinoma [13] and in colon-cancer patients with microsatellite instability–high (MSI-H) or mismatch repair–deficient (d-MMR) tumors [14].

Several studies will aim to understand which mechanisms, factors or tumor’ pathways generate inherently or acquired resistance to cancer immunotherapies [15]. Response to ICIs can be influenced by several factors: the molecular profile of cancer [16–19], histopathological features of tumor [20–22] and clinical characteristics of patient, such as site of metastases [23, 24], Eastern Cooperative Oncology Group (ECOG) Performance Status (PS) [25, 26], previous treatments [27–30] or external intervention such as drug interactions with immune system [31]. While corticosteroids and antibiotics are already known to have an immunomodulatory effect [32–34], less well known is the effect of concomitant opioids therapy used in symptomatic patients for the treatment of uncontrolled pain [35].

The aim of our study is to explore the relationship between the administration of concomitant to immunotherapy drugs (such as opioids alone or in association with antibiotics/antifungals or corticosteroids), with the oncological outcomes in order to evaluate a possible negative drug interaction able to impair the immune response to anti-PD-1 / PD-L1 agents. The removal of concomitant drugs with immunoinhibitory action could play a decisive role in restoring the response to immunotherapy treatment, so improving patients' outcomes.

## Materials and methods

### Patients

This observational, multicenter, retrospective study, recruited patients with metastatic solid tumors, including NSCLC (squamous/non squamous histology), melanoma, RCC, urothelial cancer, Merkel carcinoma and colon-cancer, who have received immunotherapy from September 2014 to September 2019. The follow-up period was from October 2014 to January 2020.

Imaging evaluation based on contrast enhanced computed tomography (CT) and magnetic resonance (MRI) was performed in order to confirm the baseline disease setting and tumor burden.

Data including age, sex, body mass index (BMI), PS, comorbidities, were retrospectively collected. Primary tumor sites, previous lines of chemotherapy or target therapy and the tumor burden and the site of metastases (bone vs. visceral) were collected as well.

The concomitant intake of antibiotics, antifungals, corticosteroids and opioids were evaluated in all included patients. Based on the category of opioids, only patients receiving strong opioids were included into the analysis.

All patients provided a written informed consent, and the protocol approval of Local Ethics Committee was obtained [CE 5618].

### Treatment and assessments

Immunotherapy was administered according to the standard schedule approved for each primary tumor and line of treatment. Nivolumab was administered at the standard dose of 240 mg intra-venously at 2-weeks interval, pembrolizumab at the standard dose of 200 mg intravenously at 3-weeks interval, Atezolizumab 1200 mg at 3-weeks interval and Avelumab 800 mg at 2-weeks interval.

Imaging assessment was performed after 12 weeks or before in case of evident clinical disease progression. Tumor response was assessed using immune-related Response Evaluation Criteria in Solid Tumors (i-RECIST) [36, 37] and classified as complete response (RC), partial response (RP), stable disease (SD), and progressive disease (PD).

Treatment toxicity was assessed every 2/3 weeks, according to the National Cancer Institute-Common Terminology Criteria for Adverse Events version 4.0 (CTCAE version 4.03, 2010).

Progression-free survival (PFS) was defined as the time from patient’s first administration of ICIs until the first progression or in-treatment death. Early progression disease was defined as a progression until 3 months from the beginning of immunotherapy treatment. The OS was defined as the time from patient registration to death from any cause. Tumor burden was defined as ‘low’ (≤ 2 metastatic sites) or ‘high’ (≥ 3metastatic sites).

### Statistical analysis

In the descriptive analysis, quantitative variables were described as mean and range, while qualitative variables were reported as number and percentage. Univariate associations between clinicopathological features and opioids use were evaluated using the χ2 test. Survival curves were estimated using the Kaplan–Meier method and the log-rank test was used for the difference assessment. A multivariate Cox-proportional hazard model was used to identify independent prognostic factors for survival. Statistical significance was set at p < 0.05. SPSS statistical software, Version 25 (SPSS Inc. Chicago, Illinois, USA) was used.

## Results

### Patients

A total of 193 consecutive metastatic patients treated with ICIs in first, second line or beyond were enrolled in this study. The baseline clinical characteristics are reported in Table 1. One hundred and twenty patients (62%) were male, the median age was 70 years (range 24–91), 122 patients (63%) with less than 2 comorbidities and 94 (49%) with a good Baseline ECOG PS. The primary tumor was in 99 (51%) melanoma, in 59 (30%) NSCLC, in 28 (14%) clear cell RCC, in 5 (3%) urothelial cancer, in 1 (0.5%) Merkel carcinoma and in 1 (0.5%) colon cancer.

**Table 1:** Clinical and pathological features of the study population

	Total	OPIODS	NO OPIODS	*p*	
	N. (%)	N. (%)	N. (%)		
	193 (100)	42 (100)	151 (100)		
Sex					
Male	120 (62)	25 (60)	95(63)	0.689	
Female	73 (38)	17 (40)	56 (37)		
Age (years)					
Median					
< 65	61 (32)	14 (33)	47 (31)		
65–75	78 (40)	21 (50)	57 (38) 57 (38)		
> 75	53 (53)	7 (17)	46 (31)	0.171	
Missed	1 (1)				
Diagnosis					
NSCLC	59 (30)	21(62)	33 (22)	** < 0.0001**	
Melanoma	99 (51)	7 (17)	92 (61)		
Renal Cancer	28 (14)	5 (12)	23 (15)		
Urothelial Cancer	5 (3)	3 (7)	2 (1)		
Merkel Tumor	1 (0.5)	1 (2)	0		
Colon Cancer	1 (0.5)	0	1 (1)		
ECOG PS					
0	94 (49)	9 (21)	85 (56)		
≥1	99 (51)	33 (79)	66 (44)	** < 0.0001**	
Comorbidity					
0–1	122 (63)	29 (69)	93 (62)	0.375	
≥2	71 (37)	13 (31)	58 (38)		
Immunotherapy, drug name					
Nivolumab	121 (63)	26 (62)	95 (63)	0.145	
Pembrolizumab	60 (31)	11 (26)	49 (32)		
Atezolizumab	11 (6)	4 (10)	7 (5)		
Avelumab	1 (0.5)	1 (2)	0		
Immunotherapy setting					
First line	91 (47)	11 (26)	80 (53)		
Second line	69 (36)	21 (50)	48 (32)	**0.009**	
Beyond II-line	33 (17)	10 (24)	23 (15)		
Antibiotics/Antifungals					
Yes	21 (11)	8 (19)	13 (9)	0.055	
Not	172 (89)	34 (81)	138 (91)		
Corticosteroids					
Yes	44 (23)	20 (48)	24 (16)	** < 0.0001**	
Not	148 (77)	22 (52)	126 (84)		
Opiods					
Yes	42 (22)	-	-		
Not	151 (78)	-	-		
Tumor burden					
Low	91 (47)	12 (29)	79 (52)		
High	102 (53)	30 (71)	72 (48)	**0.006**	

*PS* ECOG performance status, *NSCLC* non-small cell lung cancer, *RCC* renal cell carcinoma

Overall, the immunotherapy treatment was planned as first line in 91 patients (46%) while 69 (37%) of patients received immunotherapy as second and 33 (17%) as subsequent lines.

Nivolumab was the most frequently prescribed drug (123 patients, 63%), followed by pembrolizumab (60 patients, 31%), while the anti PD-L1 atezolizumab and avelumab were administered in 11 (6%) patients and 1 (0.5%) patient, respectively.

Twenty-one (11%), 44 (23%) and 42 (22%) patients received antibiotics/antifungals, corticosteroids and opioids before and/or during immunotherapy (Table 1).

As it is shown in Table 1, opioids use was significantly higher in patients affected by NSCLC (*p* < 0.0001), in patients with a worse ECOG PS (*p* < 0.0001), in second-line setting subgroup (*p* = 0.009), in patients taking corticosteroids (*p* < 0.0001) and in patients with a high tumor burden (*p* = 0.006).

### Outcomes

With a median follow up of 12 months (95% CI 6.8–17.2 months), 114 (61%) disease progression and 82 (43%) deaths were reported. Early progression occurred in 101 pts (52.3%) and, considering only the concomitant medications, it was significantly associated with opioid use (*p* = 0.015) (Table 2).

**Table 2:** association between several concomitant medications and the number of early progressions

	Early PD	
	N(%)	*p*
Antibiotics/antimicotics		
YES v NOT	13 (52) v 94 (51)	1
Opioids		
YES v NOT	29 (69) v 72 (47)	0.015
Infections		
YES v NOT	8 (67) v 93 (51)	0.379

The use of opioids resulted significantly associated with early progression. In bold *p* ≤ 0.05

*PD* progressive disease

**Fig. 1:**
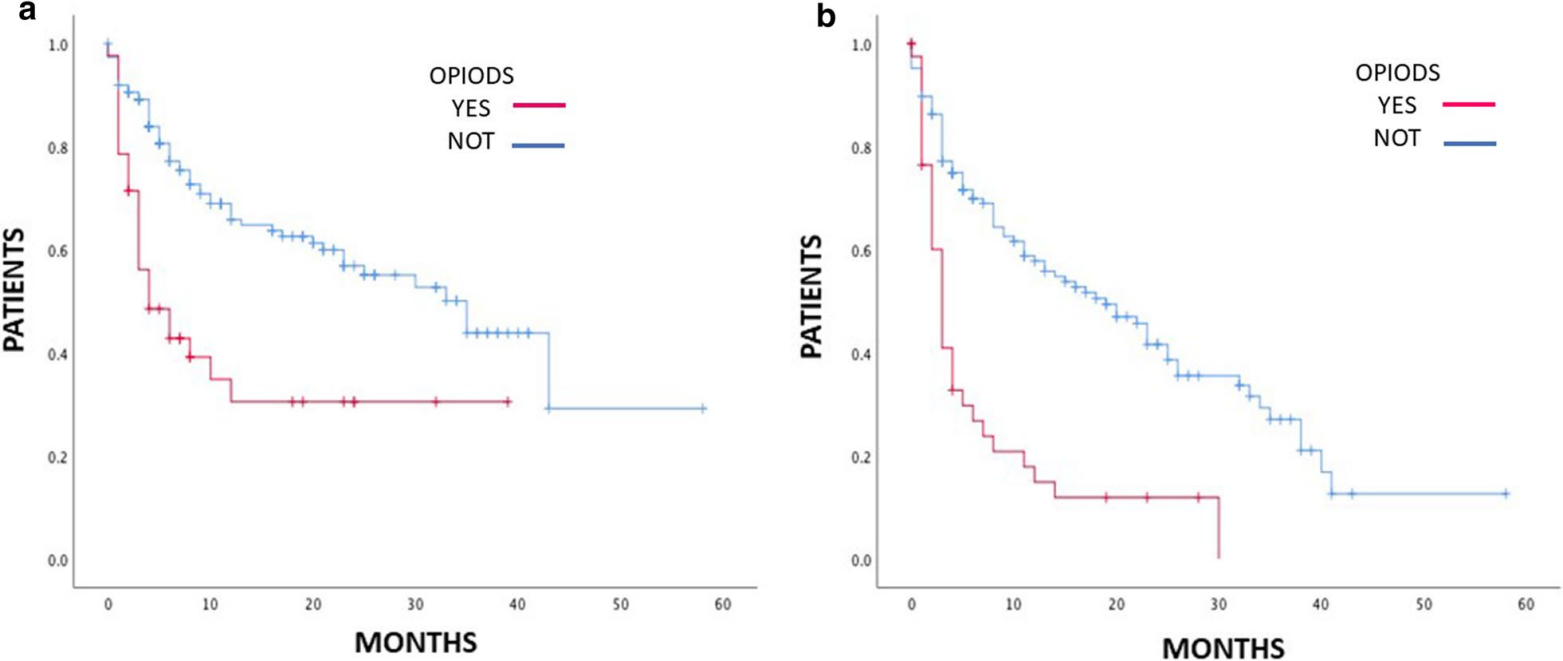
Association between opioids use and outcomes: OS (**a**) and PFS (**b**)

Overall, PFS and OS were significantly shorter in patients with an ECOG PS ≥ 1 compared to those with: ECOG PS = 0 (median PFS, 4 vs. 25 months, HR 1.65, 95% CI 1.36–1.99, *p* < 0.0001; median OS, 7 months vs. not reached, HR 2.25, 95% CI 1.74–2.90, P < 0.0001), to patients taking corticosteroids (median PFS, 3 vs. 18 months, HR 2.13, 95% CI 1.40–3.24, *p* < 0.0001; median OS, 6 vs. 35 months, HR 2.48, 95% CI 1.55–3.97, *p* < 0.0001), to patients taking opioids (median PFS, 3 vs. 19 months, HR 1.70, 95% CI 1.37–2.09, *p* < 0.0001; median OS, 4 vs. 35 months, HR 1.60, 95%CI 1.26–2.02, *p* < 0.0001, Fig. 1A/B) and patients with higher volume tumor burden (median, PFS 5 vs. 22 months, HR 1.79, 95% CI 1.22–2.62, *p* = 0.003; median OS, 10 vs. 43 months, HR 2.06, 95%CI 1.31–3.24, *p* = 0.002).

OS was significantly shorter also in patients who used antibiotics or antifungals (median OS, 6 vs. 33 months, HR 2.24, 95% CI 1.25–3.99, *p* = 0.006).

However, at the multivariate analyses, ECOG PS and opioids were independent prognostic factors for PFS (Table 3), whereas only ECOG PS resulted to be an independent prognostic factor for OS, but with trend toward significance for opioids as well as tumor burden (Table 3).

**Table 3:** Univariate and Multivariate analysis for progression free survival (PFS) and overall survival (OS)

	Cox-regression analysis for survival							
	Univariate (PFS)		Multivariate (PFS)		Univariate (OS)		Multivariate (OS)	
	HR (95%CI)	*p*	HR (95%CI)	*p*	HR (95%CI)	*P*	HR (95%CI)	*p*
Sex Female v Male	0.9 (0.60–1.30)	0.548			1.03 (0.65–1.58)	0.934		
Age categories								
> 75 v 65–75 v < 65	1.06 (0.83–1.34)	0.633			1.19 (0.89–1.58)	0.232		
Baseline ECOG PS ^3^1 v 0	1.65 (1.36–1.99)	< 0.0001	1.46 (1.19–1.80)	< 0.0001	2.25 (1.74–2.90)	< 0.0001	1.99 (1.52–2.61)	< 0.0001
Comorbidities ^3^2 v 0–1	0.84 (0.57–1.24)	0.386			0.97 (0.62–1.54)	0.918		
Immunotherapy setting III- and beyond v II- v I-line	0.956 (0.75–1.21)	0.721			1.06 (0.81–1.42)	0.606		
Antibiotics antimicotics								
YES v NOT	1.5 (0.82–2.73)	0.187			2.24 (1.25–3.99)	0.006	1.48 (0.80–2.73)	0.201
Corticosteroids								
YES v NOT	2.13 (1.40–3.24)	< 0.0001	1.42 (0.90–2.23)	0.122	2.48 (1.55–3.97)	< 0.0001	1.41 (0.84–2.35)	0.19
Opioids								
YES v NOT	1.69 (1.37–2.09)	< 0.0001	1.44 (1.15–1.79)	0.001	1.6 (1.26–2.02)	< 0.0001	1.24 (0.97–1.61)	0.087
Tumor burden								
High v Low	1.79 (1.22–2.62)	0.003	1.43 (0.97–2.11)	0.071	2.06 (1.31–3.24)	0.002	1.58 (0.98–2.52)	0.057

*mPFS *median progression free survival, *mOS* median overall survival, *HR* hazard ratio, *p* p value. In bold p ≤ 0.05

**Fig. 2:**
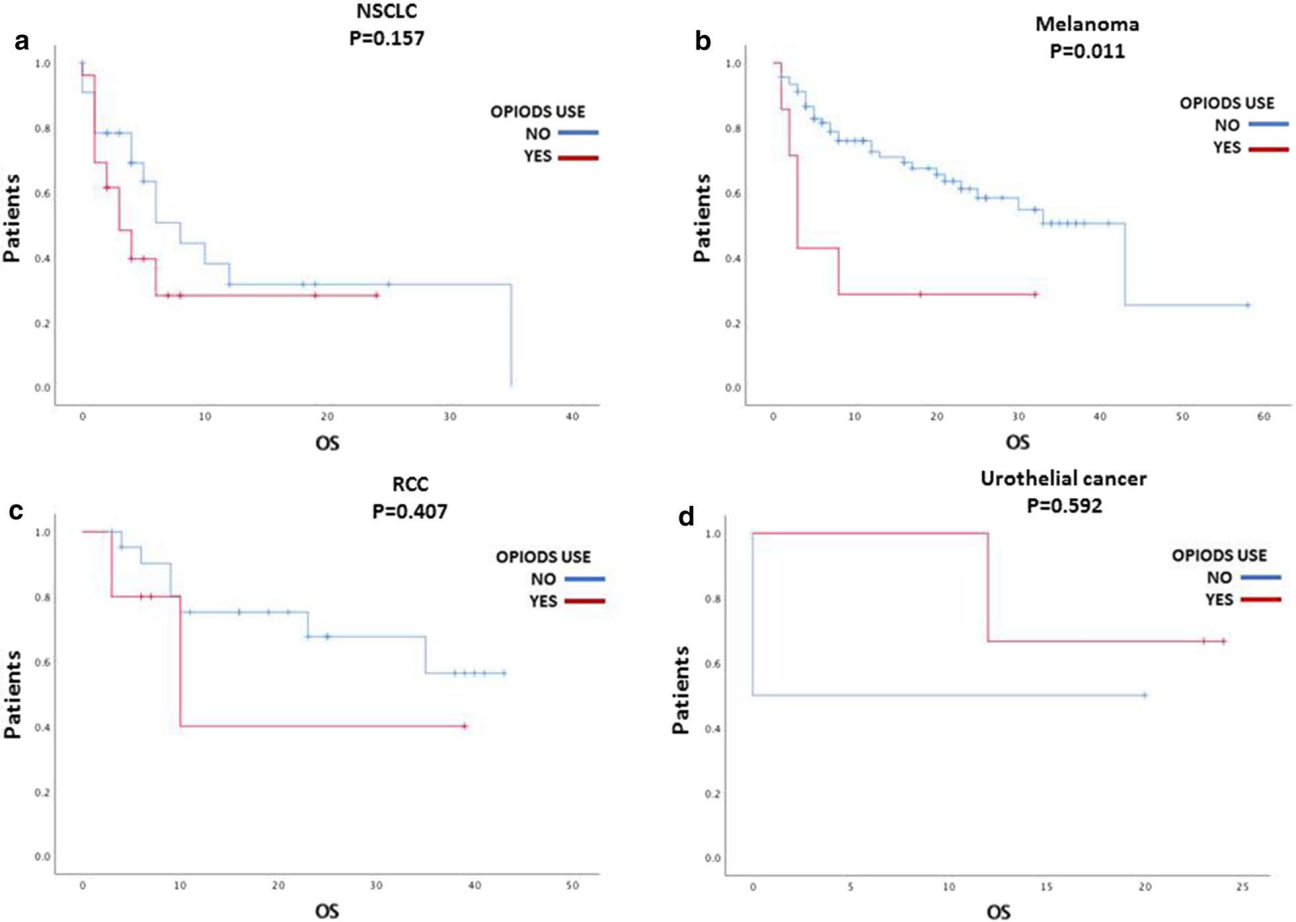
Impact on survival of opioids in different types of cancer. **a** Non small cell lung cancer (NSCLC), **b** melanoma, **c** renal cell carcinoma (RCC). **d** Urothelial cancer

In these analyses for survival we didn’t include primary tumor diagnosis among clinicopathological factors examined, according to the different tumor-intrinsic prognosis which does not make a direct comparison possible. As shown in Fig. 2, there is a statistical significance between opioids use and survival only in melanoma subgroup (*p* = 0.011).

**Fig. 3:**
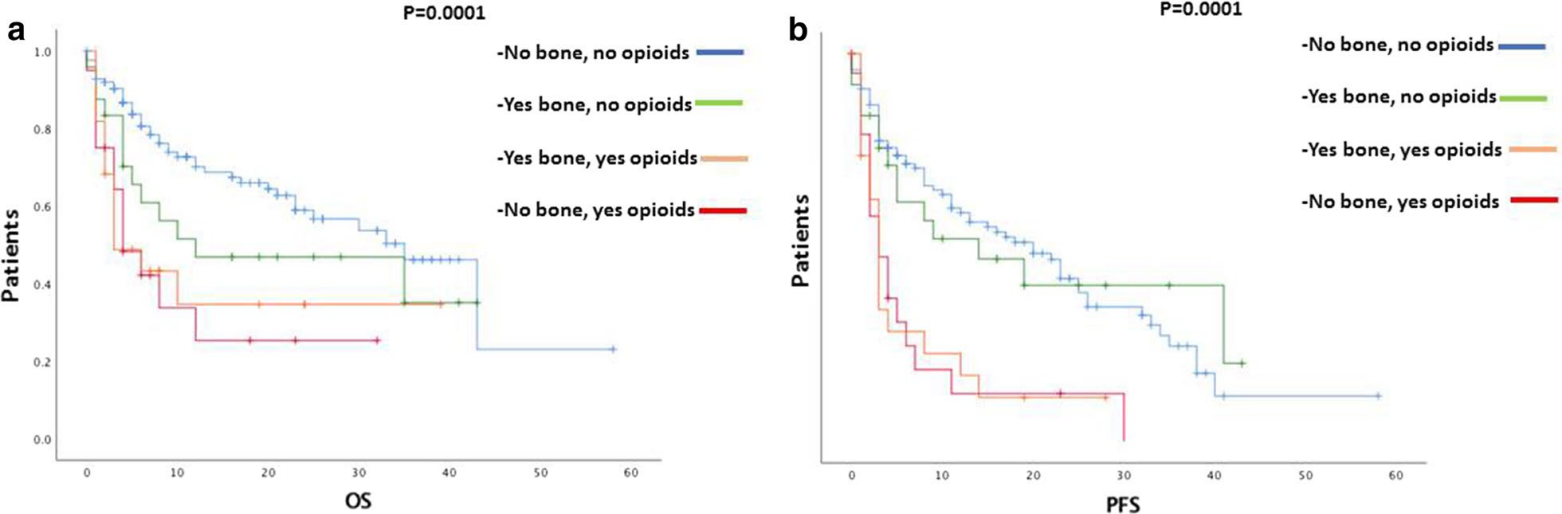
Impact on survival outcomes of opioids in patients with bone metastases. Oncological patients who need opioids during immunotherapy have worse OS (**a**) and PFS (**b**) regardless of they have or have not bone metastases

Both OS and PFS were significantly shorter in patients taking opioids regardless of the presence of bone metastases (*p* < 0.0001) [Fig. 3].

## Discussion

Despite the success of immunotherapy in the cancer treatment, only a small percentage of patients presents long term benefit. So, the research of biomarkers represents an urgent need considering that only PD-L1 is routinely available to choose the treatment strategy of our patients. In this context, clinical features could drive the physicians for the definition of therapeutic strategy.

Our study, including different solid tumors, suggests that some clinical features such as ECOG PS and concomitant administration of opioids could negatively predict the outcomes of cancer patients receiving immunotherapy. In particular, opioids use is associated with early progression and could represent a predictive factor for PFS. Moreover, on multivariate analysis, the use of opioids appears to have a tendency to negatively influence OS as well.

ECOG PS has been confirmed to be one of the most important prognostic factors, indeed a worse PS is closely associated with high tumor burden and symptomatic disease requiring concomitant therapies. Corticosteroids are well known to have an immunosuppressive action however they are used for the treatment of adverse immune-related events of immunotherapy [37]. Indeed, corticosteroids are able to activate the glucocorticoids responsive elements (GRE) resulting in a inhibition in IL-1 and IL-6 transcription and in a reduction in T cell function [38].

On the other hand, also the link between opioids and immune system could play a crucial role to determine the resistance to immunotherapy due to the presence of opioids receptor on immune cells.

Indeed, it has been shown, on mice spleen models, the presence of μ receptors on lymphocytes surface and in vitro experiments that the administration of morphine affected directly the lymphocytes proliferation and antibody formation, by binding to μ receptors [39–41].

Furthermore, morphine and buprenorphine, through the p38 MAPK and the calcium pathway, with a mechanism ligand dependent, induced substantial reduction of interleukin-4 mRNA and protein in T cells [42].

While methadone by acting on μ and δ receptors on lymphocytes is able to limit the immune system response, in vitro studies showed that at the transcription level this analgesic drug can decrease the proliferation and the activity of lymphocytes through down-regulation of G-protein- coupled opioid receptor gene. The consequent DNA methylation can suppress immune function [43].

It was pointed out that morphine decreases the ability of natural killer (NK) cells and in particular to induce apoptosis in a target tumor cell line, through both the classical opioid receptor and Toll-like receptor (TLR)-4 [44]. These studies, using purified primary human NK cells from peripheral blood and opioid receptor- or TL4 pathway-specific inhibitors, have shown that morphine appears to increase NK cell secretion of IL-6, granzyme A, and granzyme B. This production was so copious and unbalanced that cytotoxic efficiency of immune system was compromised [45].

It has been studied the role of Fentanyl in the perioperative period especially after 48 h after surgery, pointing out that when administered with large dose anesthesia, caused a suppression of NK cell function. The related mechanism though which this occurs consists in the impairment of the activity of the hypothalamic–pituitary–adrenal (HPA) axis, resulting in lower levels of adrenocorticotropic hormone and cortisol or reduction in the production of cytokines such as IFN-y and tumor necrosis factor-α (TNFα) [46].

Several studies investigated the role of opioid receptors on lymphocytes surface and their ability, after the binding with an agonist, to reduce the activity of the immune system. It has been proven that in addition to the three classical opioid receptors μ, k and δ, a fourth receptor is involved namely N/OFQ peptide receptor (NOP). This is present on several immune cell subtypes such as polymorphonuclear cells, B cells, T cells and monocytes and mast cells. Even if with little affinity, morphine binds to the NOP with the consequent inhibition of release of immunomodulatory neurotransmitters such as dopamine, histamine, noradrenaline and glutamate resulting in a reduction of immune activity [47].

Moreover, clinical and preclinical evidences suggest that opioids drugs are able to modify the GUT microbiota inducing microbial dysbiosis and bacterial translocation through the impairment of the mucosal barrier function. These changes in gut microbiota could trigger inflammation and abnormal immune response [48–50].

In literature, there are few clinical evidences about the effect of opioid use in cancer response to immunotherapy. In a retrospective study including 102 patients with advanced cancer in treatment with immunotherapy, antibiotic and opioids use were associated with poor outcome in term of PFS and OS [51]. To our knowledge, our study population is the most numerous among studies aimed at investigating the relationship between opioid therapy and outcomes during immunotherapy. Although the negative prognostic impact of bone metastases during immunotherapy is confirmed in literature, our results highlighted that patients with bone metastases taking opioids have the worst prognosis regardless all other site of metastasis [52–54], highlighting the prognostic independence of opioid-based therapy from prevalent metastatic site.

However, our study has several limitations due to its retrospective nature; it includes an heterogeneous population in terms of primary tumor, line of therapy, and kind of anti PD1/PD-L1 agent administered. Moreover, the use of analgesic treatment is more frequently used in patients with advanced cancer, in compromised general condition by the burden of disease and symptoms that all could act as possible confounding factors to the retrospective analysis. All patients in study population received strong opioids including morphine, fentanyl and oxycodone. Given the restrospective nature of the study, it was not possible to define the impact of the specific opiod on survival since most patients underwent opioid switch during immunotherapy, also experimenting with different dosages. The current clinical practice of opioids rotation would have created numerous biases in the retrospective analysis. It is desirable, given the strong biological rationale demonstrated, to conduct prospective studies to explore the impact of opioids on immune system modulation possibly trying to differentiate the actions and consequences of the different types of opioid drugs.

Moreover, concomitant poly-pharmacological therapies identify a class of patients characterized by worse general clinical conditions, heavily pre-treated, with a high burden of disease and comorbidities with a consequently a poor prognosis group so as to expect a poor outcome from immunotherapy.

## Conclusion

In conclusion, before starting immunotherapy each patient should undergo to an overall multidisciplinary assessment in order to organize a safe therapeutic approach by identifying all the clinical aspects that may compromise the outcomes. A correct clinical evaluation together with new predictive molecular biomarkers will allow in the future to a better selection of patients and the personalization of treatments removing negative drug interactions and finally by applying the principle of precision medicine.

## Data Availability

The datasets used and/or analysed during the current study are available from the corresponding author on reasonable request.

## Abbreviations

ICIs: Immune checkpoint inhibitors
OS: Overall survival
PDL1: Programmed death ligand-1
NSCLC: No small cell lung cancer
RCC: Renal cell carcinoma
d-MMR: Mismatch repair–deficient
MSI-H: Microsatellite instability–high
ECOG: Eastern Cooperative Oncology Group
PS: Performance status
CT: Computed tomography
MRI: Magnetic resonance
BMI: Body mass index
i-RECIST: Immune-related Response Evaluation Criteria in Solid Tumors
RC: Complete response
SD: Stable disease
PD: Progressive disease
PFS: Progression free survival
CTCAE: Common Terminology Criteria for Adverse Events version
GRE: Glucocorticoids responsive elements
NK: Natural killer
TLR: Toll like receptor
HPA: Hypothalamic–pituitary–adrenal
TNFα: Tumor necrosis factor α
NOP: N/OFQ peptide receptor
